# Supplementary material for: Quantifying the local adaptive landscape of a nascent bacterial community
Source: Nat Commun. 2023 Jan 16;14:248. doi: 10.1038/s41467-022-35677-5 (PMC9842643; doi:10.1038/s41467-022-35677-5)
Supplement: Supplementary file 1 — Supplementary Information [file 41467_2022_35677_MOESM1_ESM.pdf]

Quantifying the Local Adaptive Landscape of a Nascent Bacterial Community

Joao A Ascensao, Kelly M Wetmore, Benjamin H Good, Adam P Arkin, and Oskar Hallatschek

Supplementary Figures

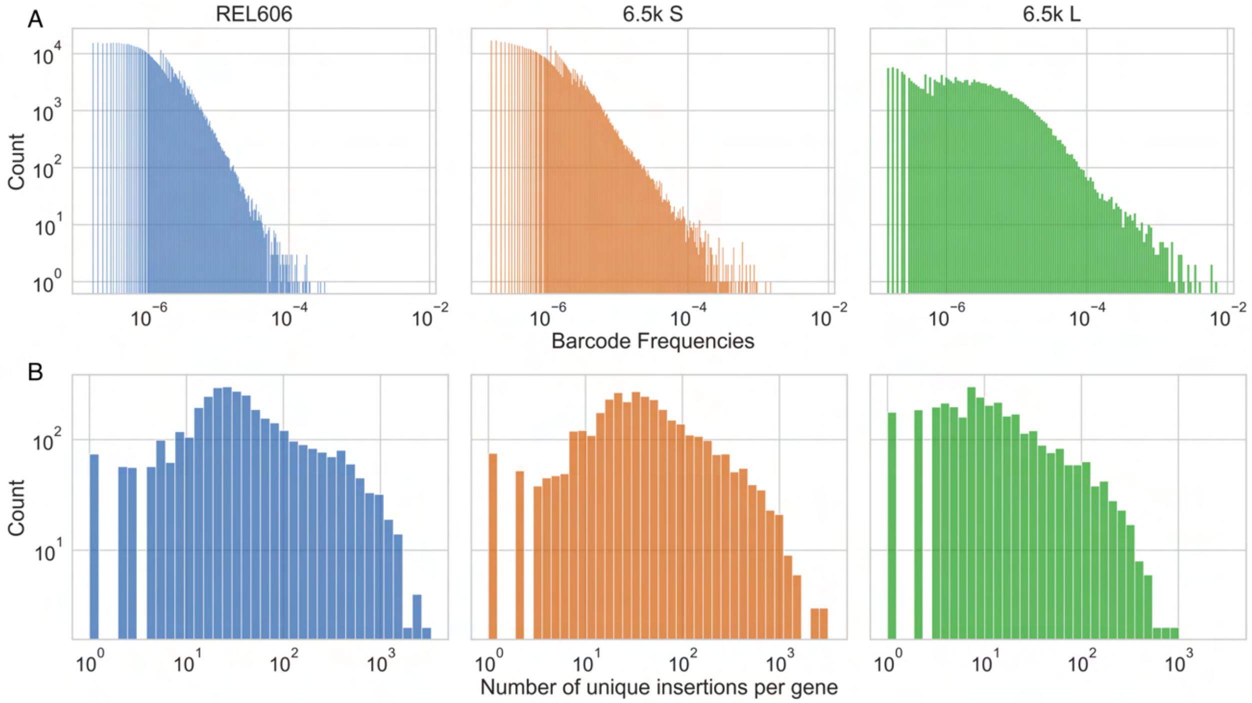

Fig. S1. Statistics of RB-TnSeq libraries, (**A**) initial distribution of barcode frequencies in library populations, and (**B**) distribution of number of unique barcoded transposon insertions into each gene (cds).

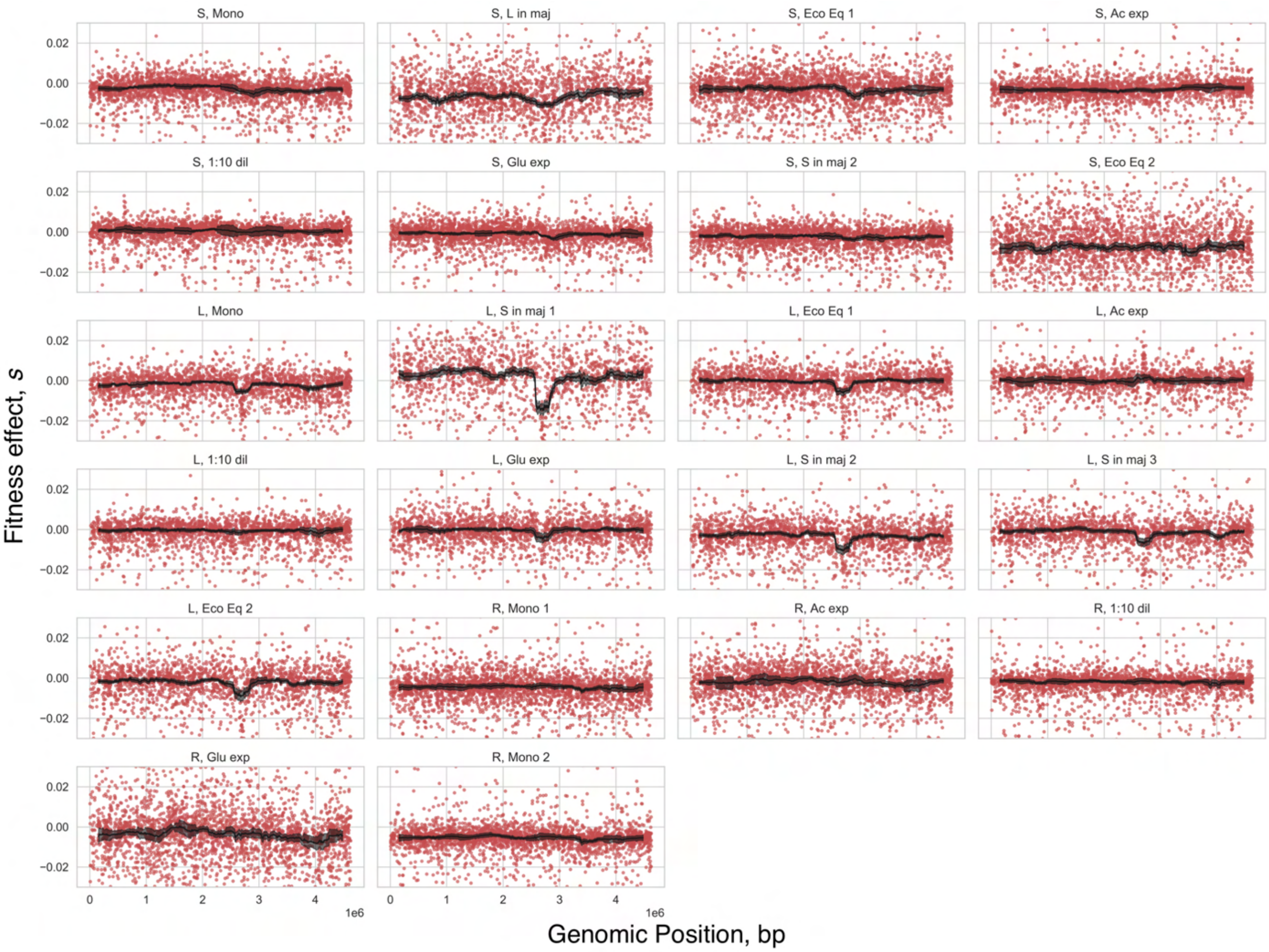

Fig. S2. Relationship between genomic position and fitness effect. Red dots are the fitness effects of individual knockouts, black line is the rolling median fitness effect (error bars are standard errors).

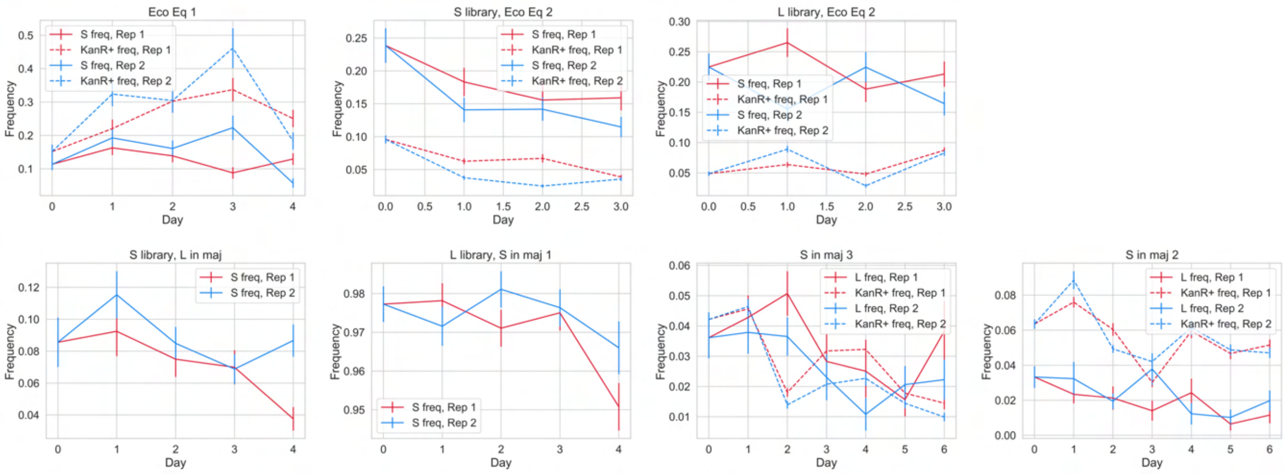

Fig. S3. Frequency trajectories of mixed culture experiments from CFUs. For each coculture experiment, we diluted and plated cultures on both TM plates (S/L indicator plates) and LB/Kan plates (pulls out cells from the RB-TnSeq libraries). We didn't plate experiments "S/L in maj (1)" on LB/Kan plates because we only cocultured wt S/L with L/S RB-TnSeq libraries respectively. Please note that each subplot is on a different scale. Points are empirical frequencies ± standard errors, calculated from binomial counting statistics.

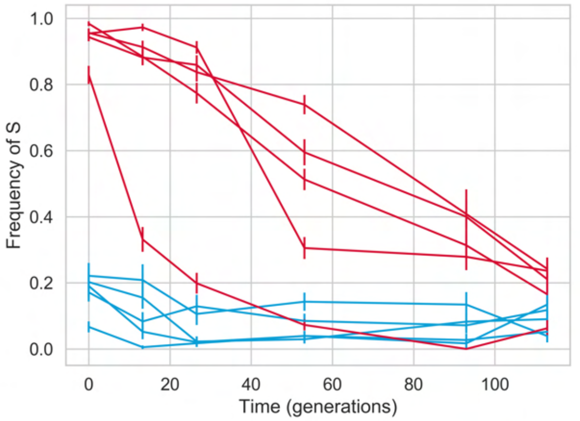

Fig. S4. Measured S/L frequency dependent fitness and ecological equilibrium via CFUs on TM plates (S/L indicator plates). Independent cocultures of S and L wt clones were propagated in standard LTEE conditions. Red lines indicate cultures that were started at high frequencies of S, blue lines indicate cultures started at low frequencies. Error bars represent standard errors, calculated from binomial counting statistics.

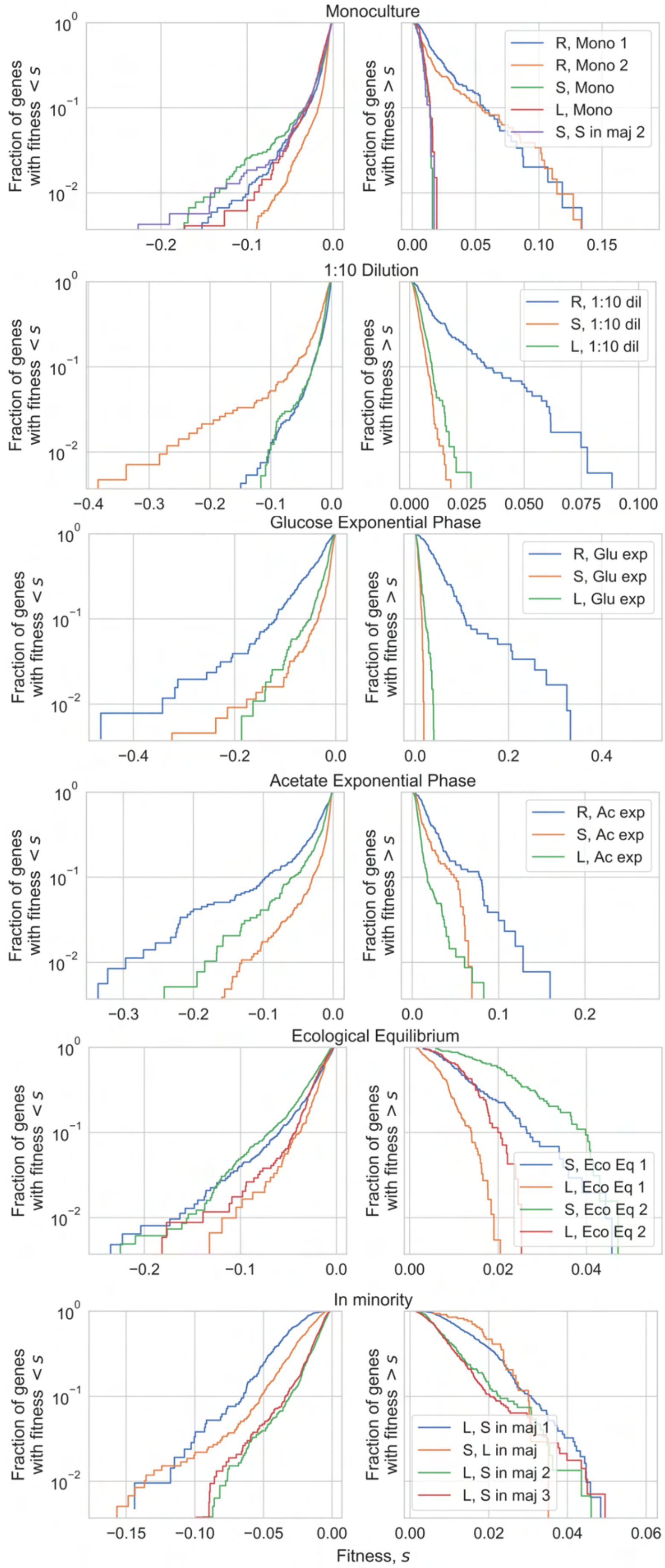

Fig. S5. All measured DFEs across experiments, arranged by environment.

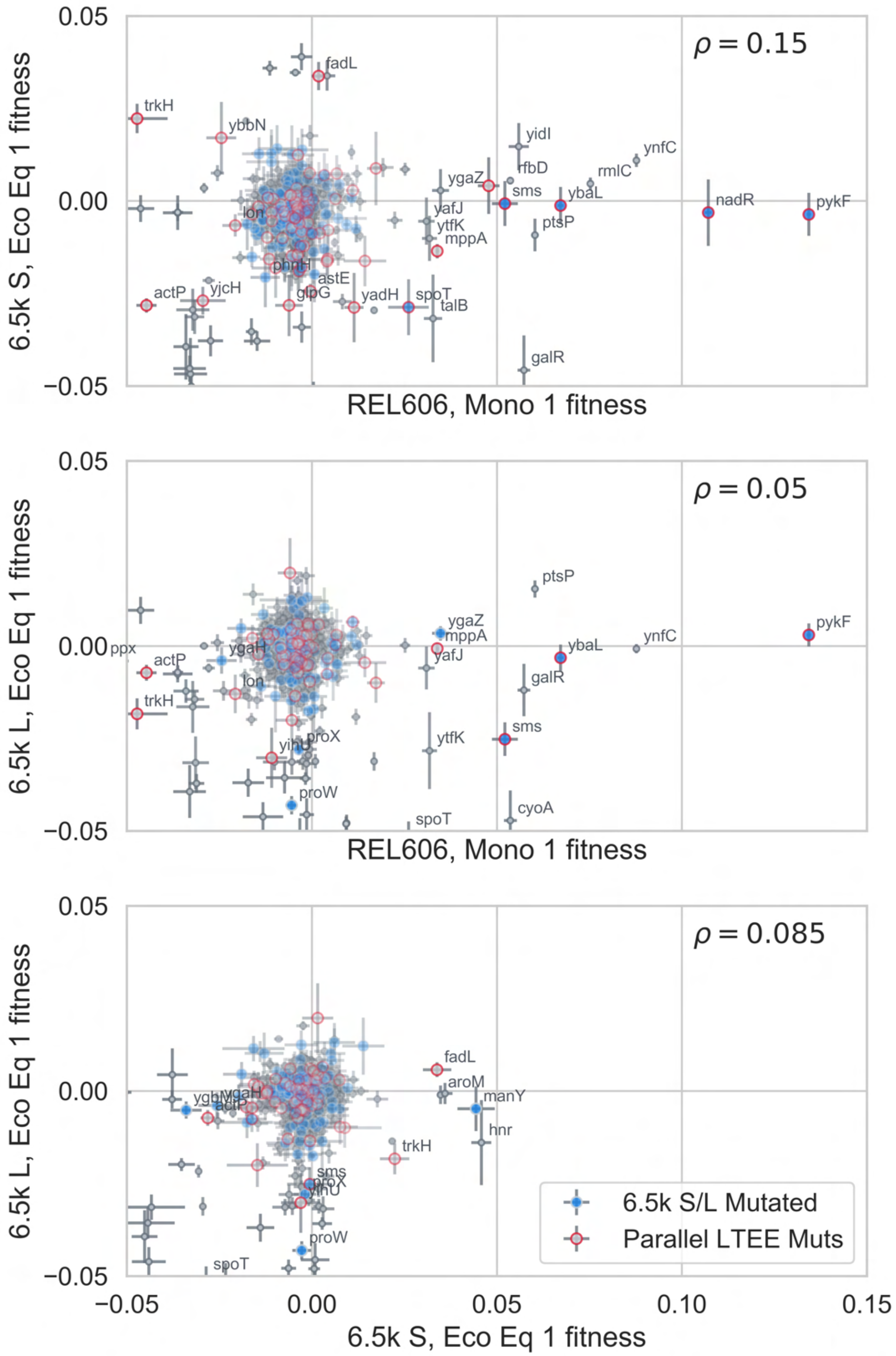

Fig. S6. Comparison of fitness effects; identical to Figure 1F in the main text, except we highlighted all genes in mutated operons. It is still the case that there are many genes that did not get a mutation in their operon, but still changed from a beneficial to non-beneficial fitness effect across genetic backgrounds. Error bars represent standard errors, as calculated in methods section 3.2.

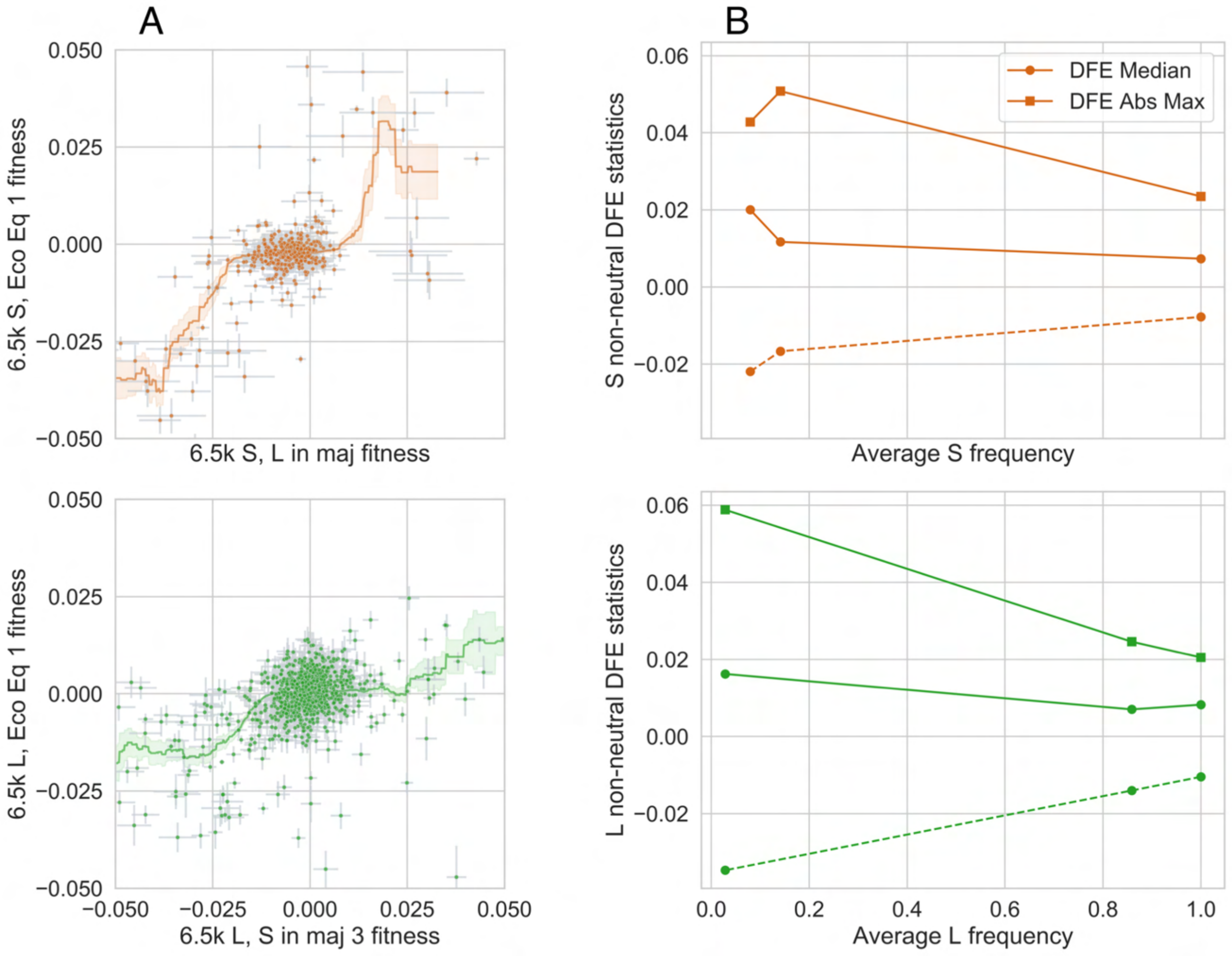

Fig. S7. Frequency-dependent knockout fitness effects for both 6.5k S and L. (**A**) Similar to Fig 2A in main text, except comparing fitness at ecological equilibrium to fitness when the ecotype is in the minority. Line shown is a rolling average of fitness effects $\pm$ standard error. Error bars on points represent standard errors, as calculated in methods section 3.2. (**B**) Changes in summary statistics of the DFE as a function of ecotype frequency. Solid lines represent the beneficial side of the DFE, while dashed lines represent the deleterious side.

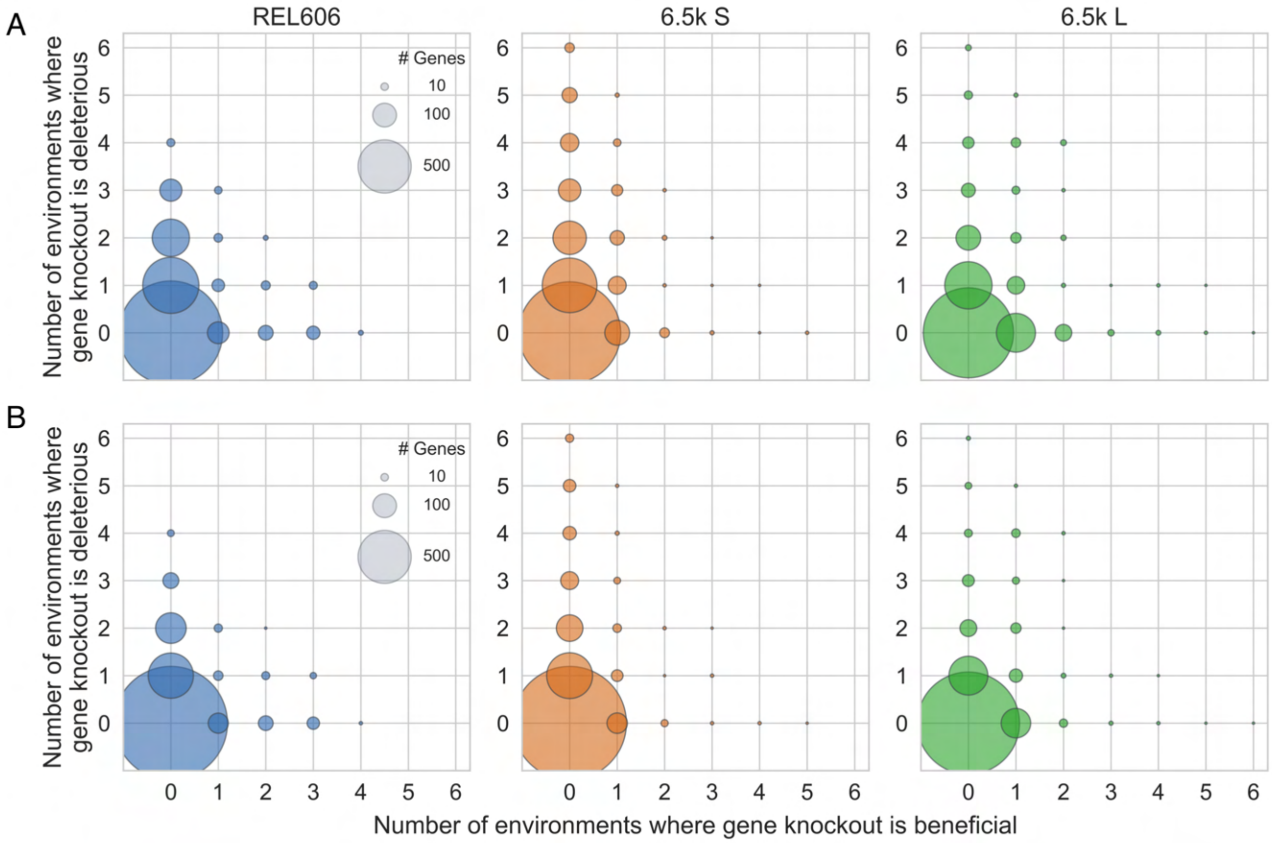

Fig. S8. Fitness effect sign-flipping across environments. Same as Figure 2C (main text), but (post-FDR correction) p-value cutoff is reduced from 0.05 to (**A**) 10^{-3} or (**B**) 10^{-5}. P-values were calculated as described in methods section 3.2.

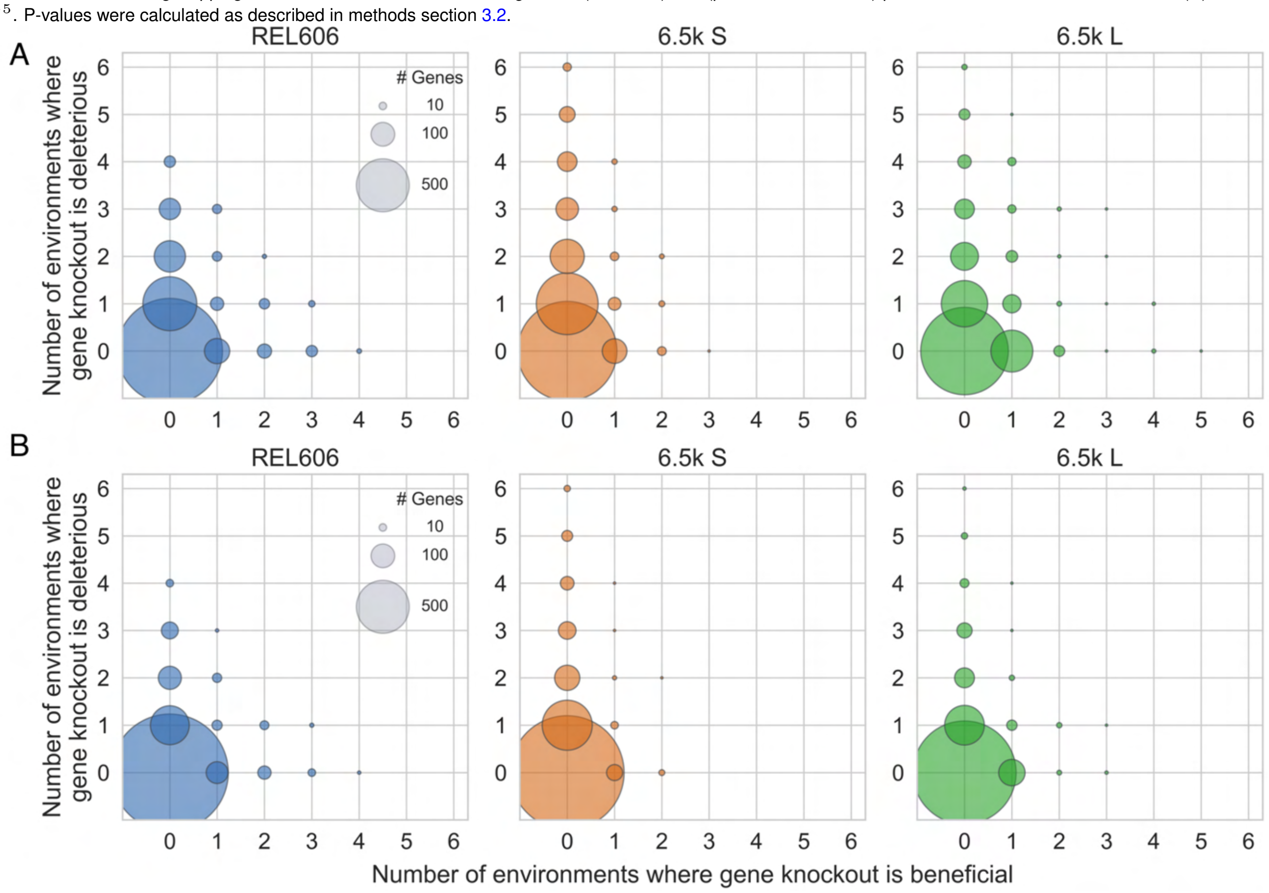

Fig. S9. Fitness effect sign-flipping across environments. Same as Figure 2C (main text), but we only consider genes non-neutral with fitness (**A**) $|s| > 1\%$ or (**B**) $|s| > 2\%$. P-values were calculated as described in methods section 3.2.

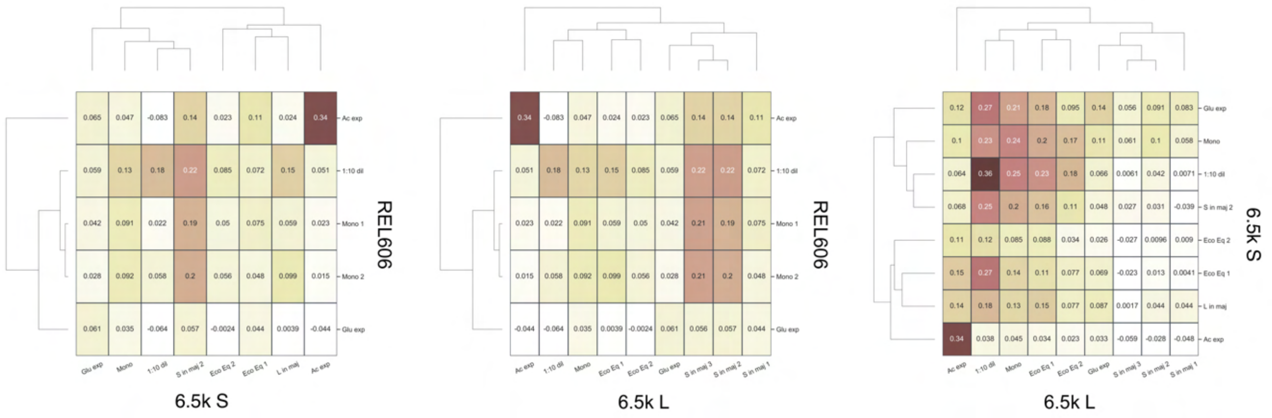

Fig. S10. Fitness effect correlations between strains.

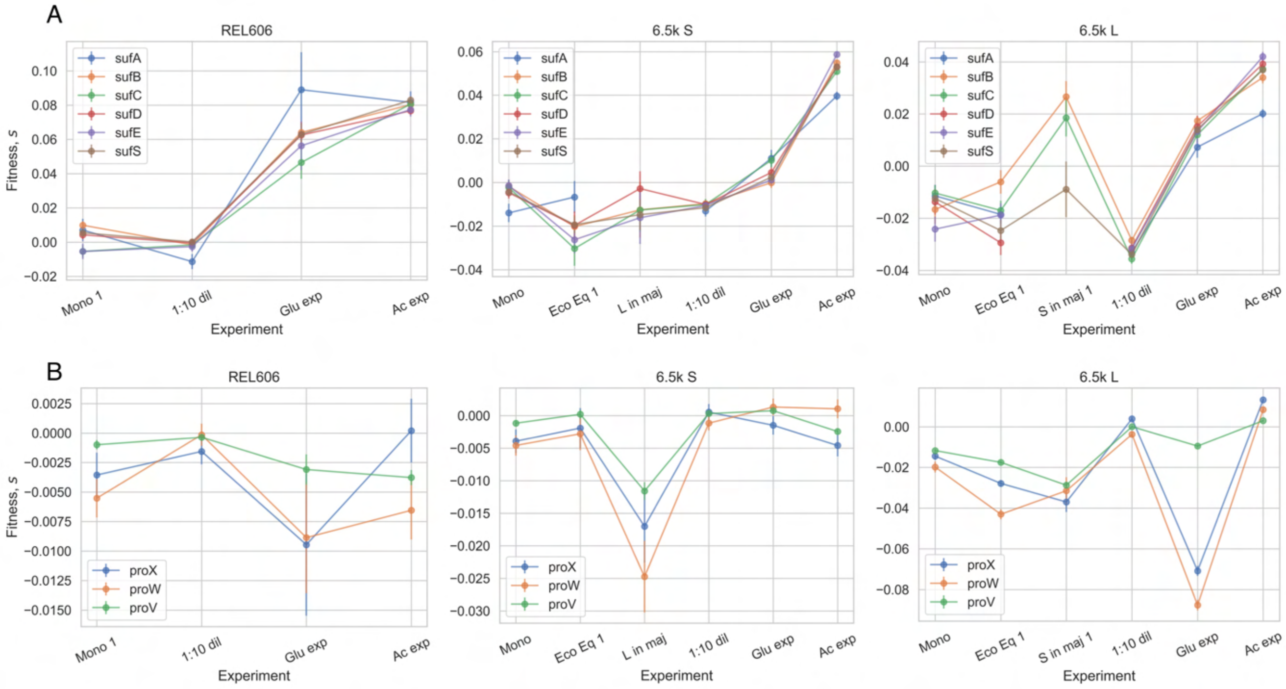

Fig. S11. Fitness effects of (**A**) *sufABCDSE* and (**B**) *proVWX* operons in REL606 and 6.5k L/S. Error bars represent standard errors, as calculated in methods section 3.2.

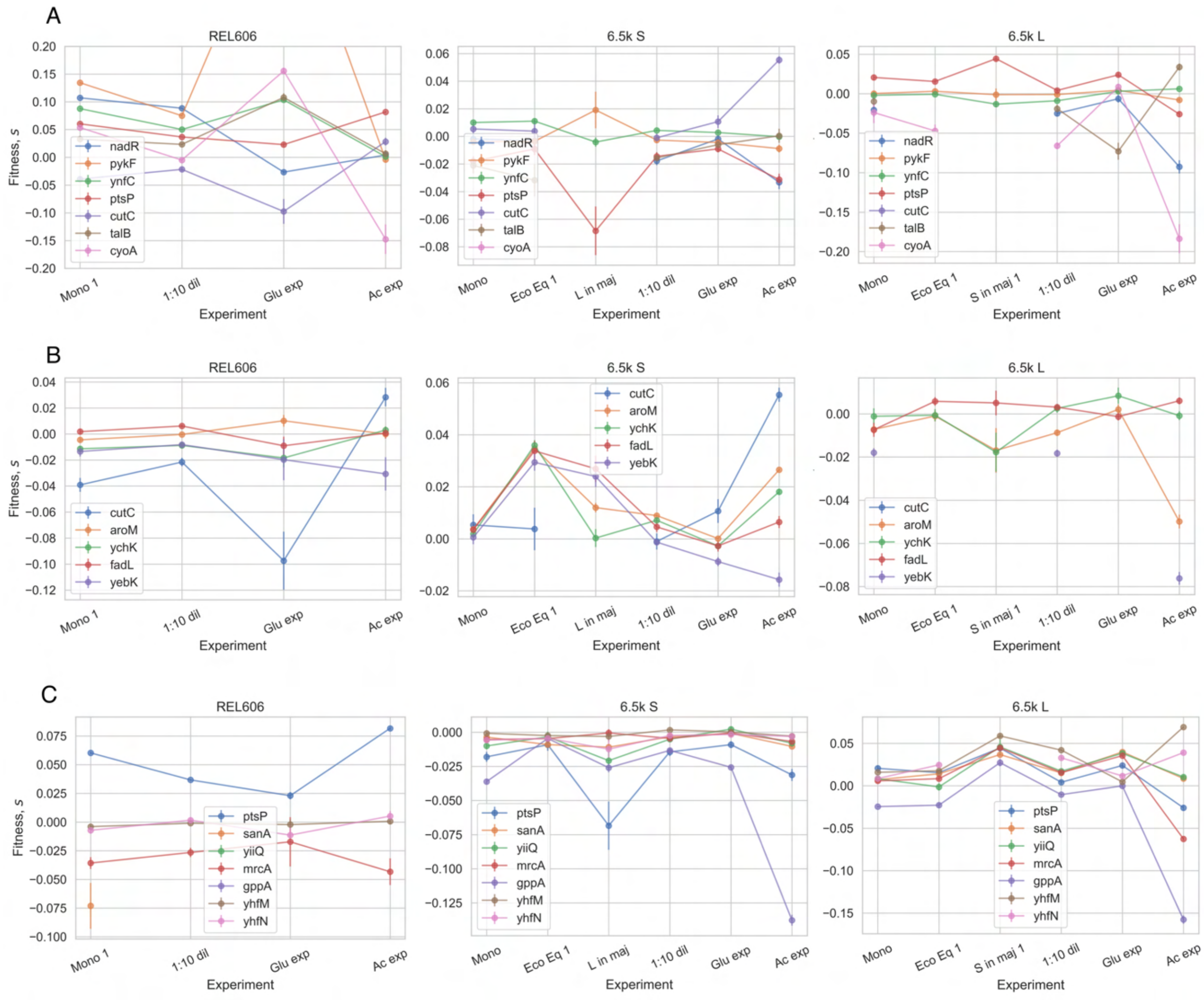

Fig. S12. Fitness effects of knockouts across environments, where knockouts are beneficial in at least one condition on the (**A**) REL606, (**B**) 6.5k S, (**C**) 6.5k L background. Error bars represent standard errors, as calculated in methods section 3.2.

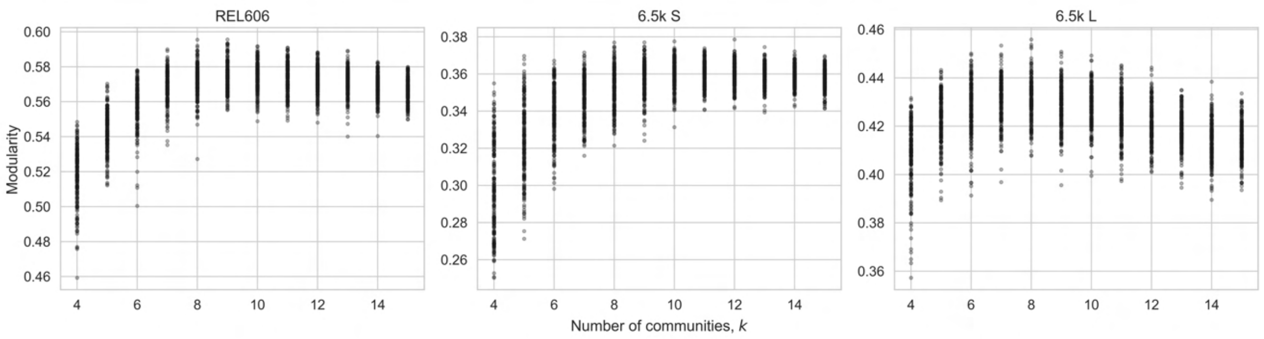

Fig. S13. Modularity of cofitness clusters, across 200 (stochastic) initializations for different numbers of communities from $4 - 15$.

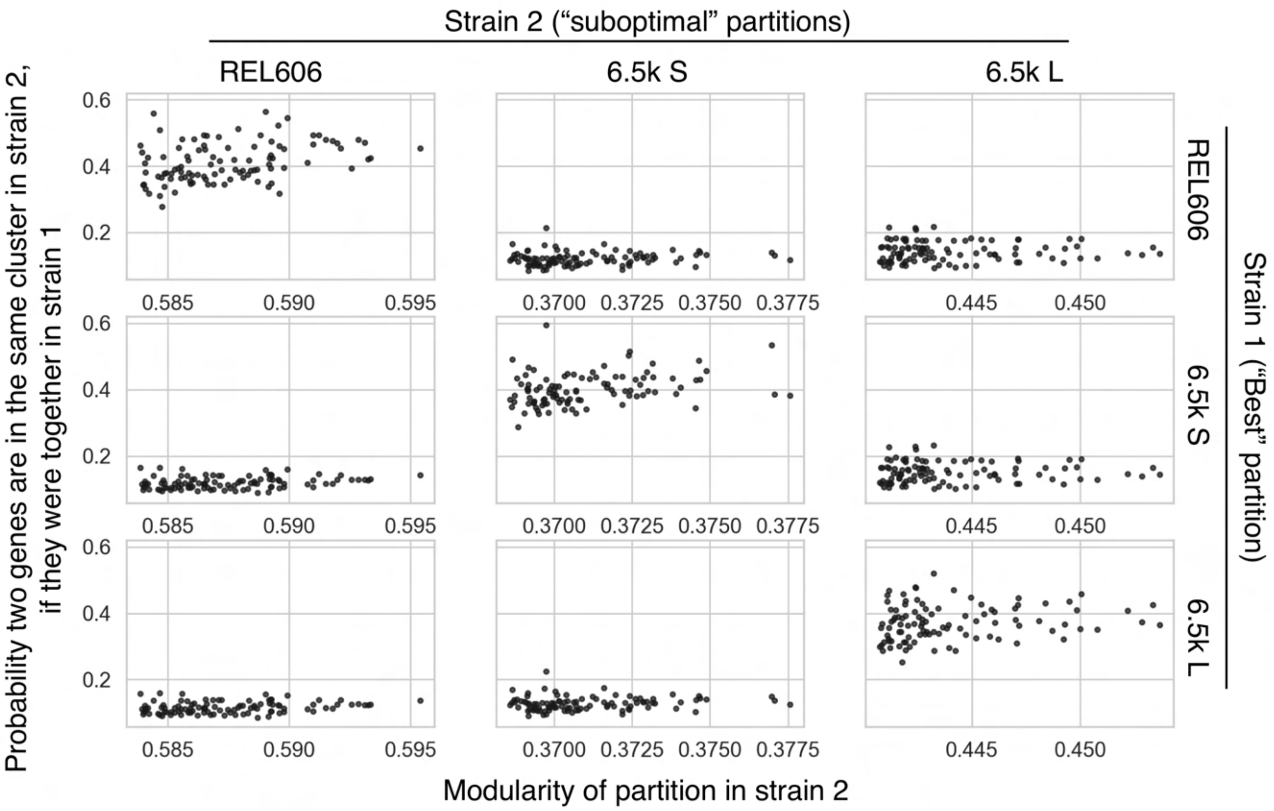

Fig. S14. We compared the optimal partition of the REL606/S/L cofitness networks to the next 100 best (but suboptimal) partitions, also shown in Figure S13. For each suboptimal partition, we asked if two genes were in the same cluster in the optimal partition, what is the probability that they are also in the same cluster in the suboptimal partition. We can see that if we compare partitions in the same genetic background, this probability is around 40%, while it is around 10% when comparing partitions across background. This suggests that different reasonable partitions of the cofitness networks are much more similar within genetic backgrounds than between backgrounds.

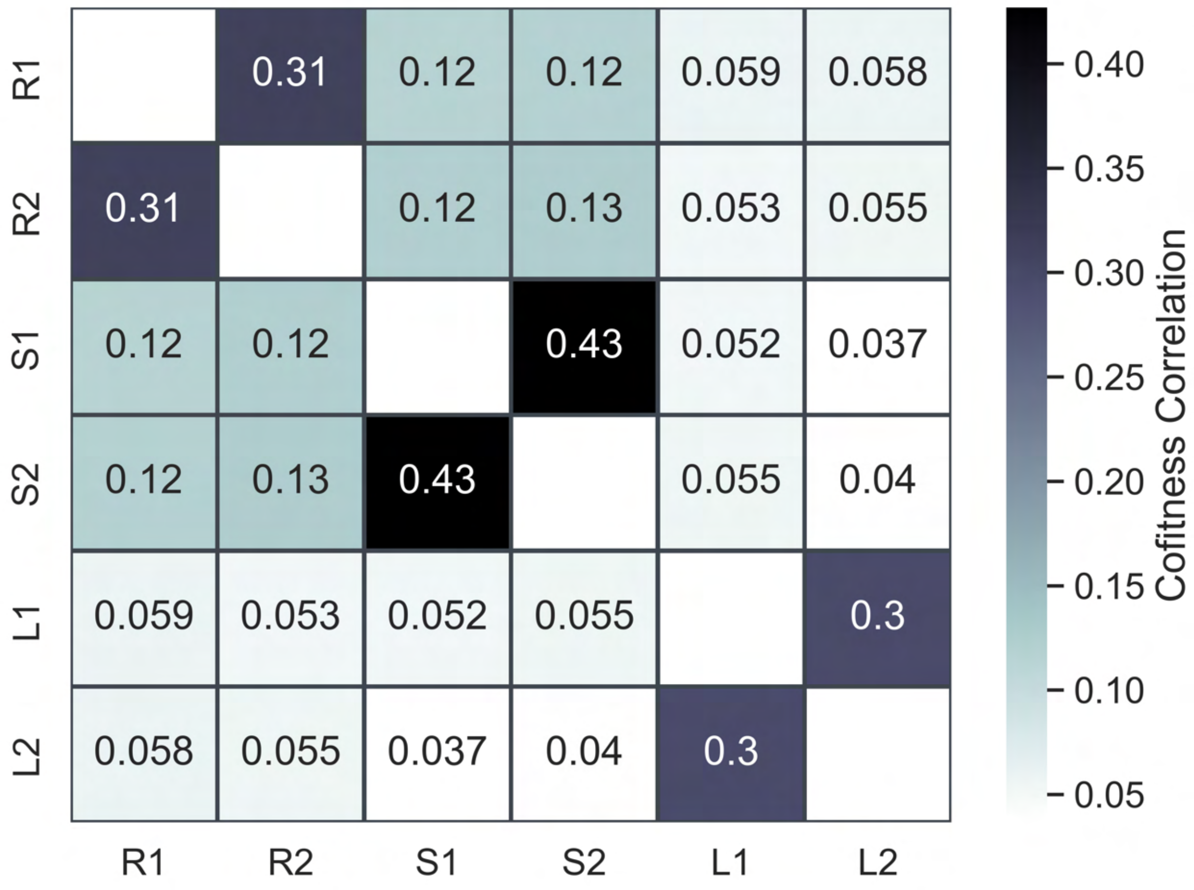

Fig. S15. In order to better understand the extent to which the structure of our cofitness networks is driven by measurement noise, we re-computed the cofitness networks, only using one of the biological replicates per experiment for every experiment. We then computed the correlation of all cofitness values across all networks. We can see that even when the data is independently split, the cofitness networks within a genetic background are more similar than between backgrounds. In the figure, R, S and L refer to REL606, 6.5k S/L libraries, respectively, and 1 and 2 refer to using only biological replicates "1" and "2" from each experiment.

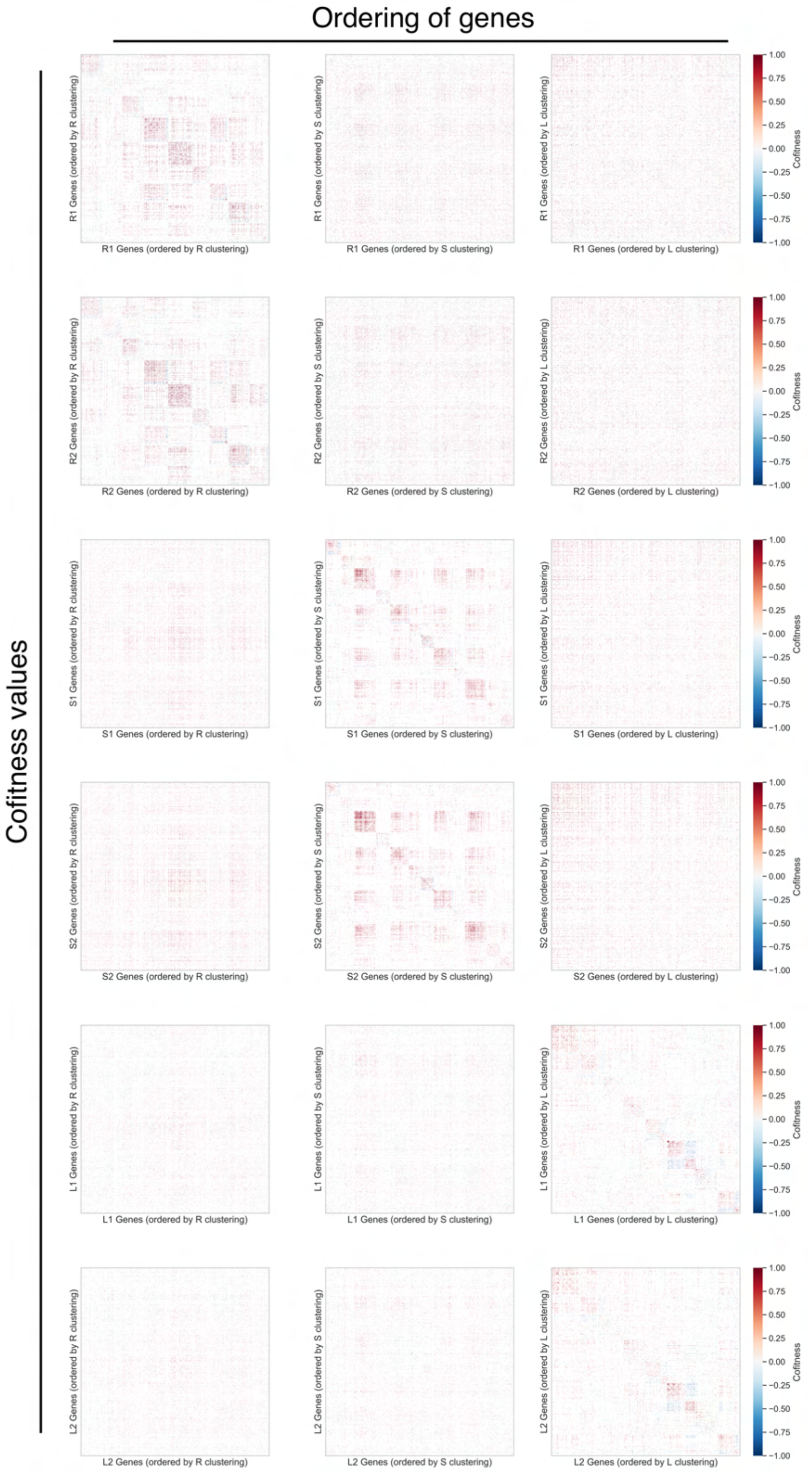

Fig. S16. We repeated the cofitness clustering, as done in Figure 4B, using cofitness networks computed using only one of the biological replicates per experiment for every experiment (as done in Figure S15). We see similar results to Figure 4B, where clusters are visibly preserved only when clustered on the same background, albeit to a weaker extent. In the figure, R, S and L refer to REL606, 6.5k S/L libraries, respectively, and 1 and 2 refer to using only biological replicates "1" and "2" from each experiment.

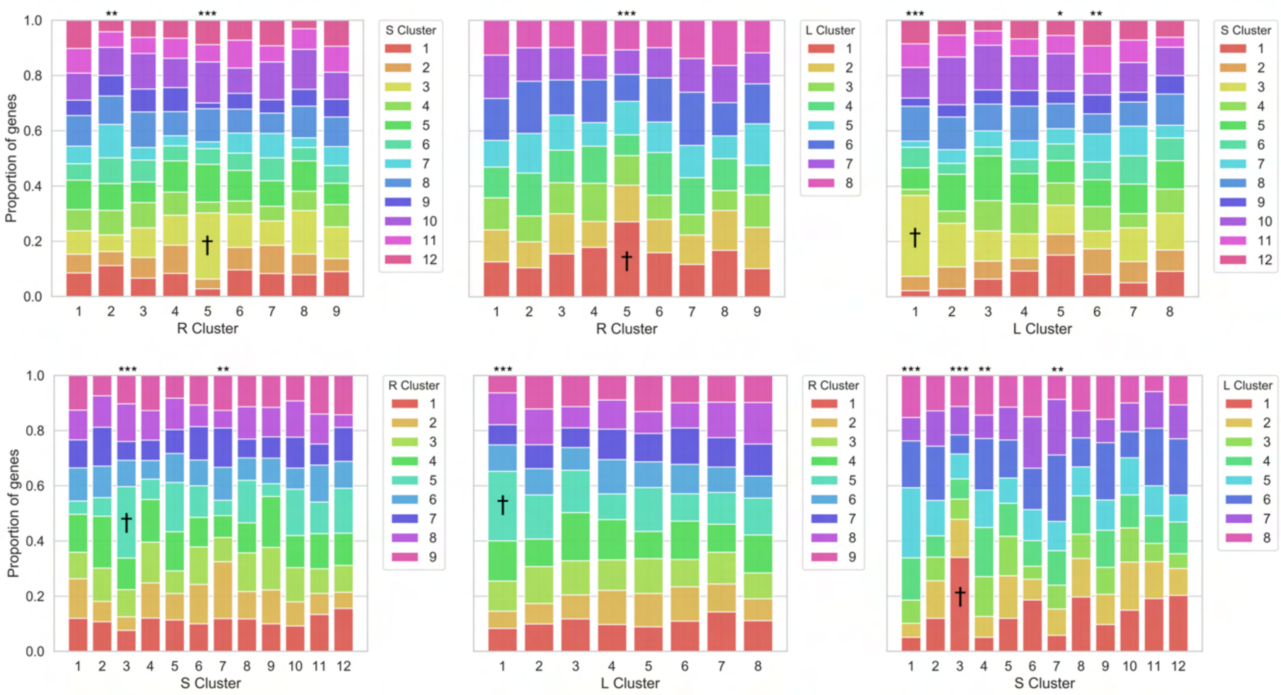

Fig. S17. In order to explore how clusters of genes changed across genetic background, we calculated the fraction of genes in a given cluster that belong to a cluster in a different genetic background. We see that clusters are mostly not preserved between genetic backgrounds, with the exception of the clusters marked by asterisks, which show non-random sampling across genetic backgrounds (* $p < 0.05$, ** $p < 0.005$, *** $p < 10^{-4}$; Pearson's chi-squared test). †set of clusters across all three genetic backgrounds which share more genes than expected, driven primarily by adhesion-related genes.

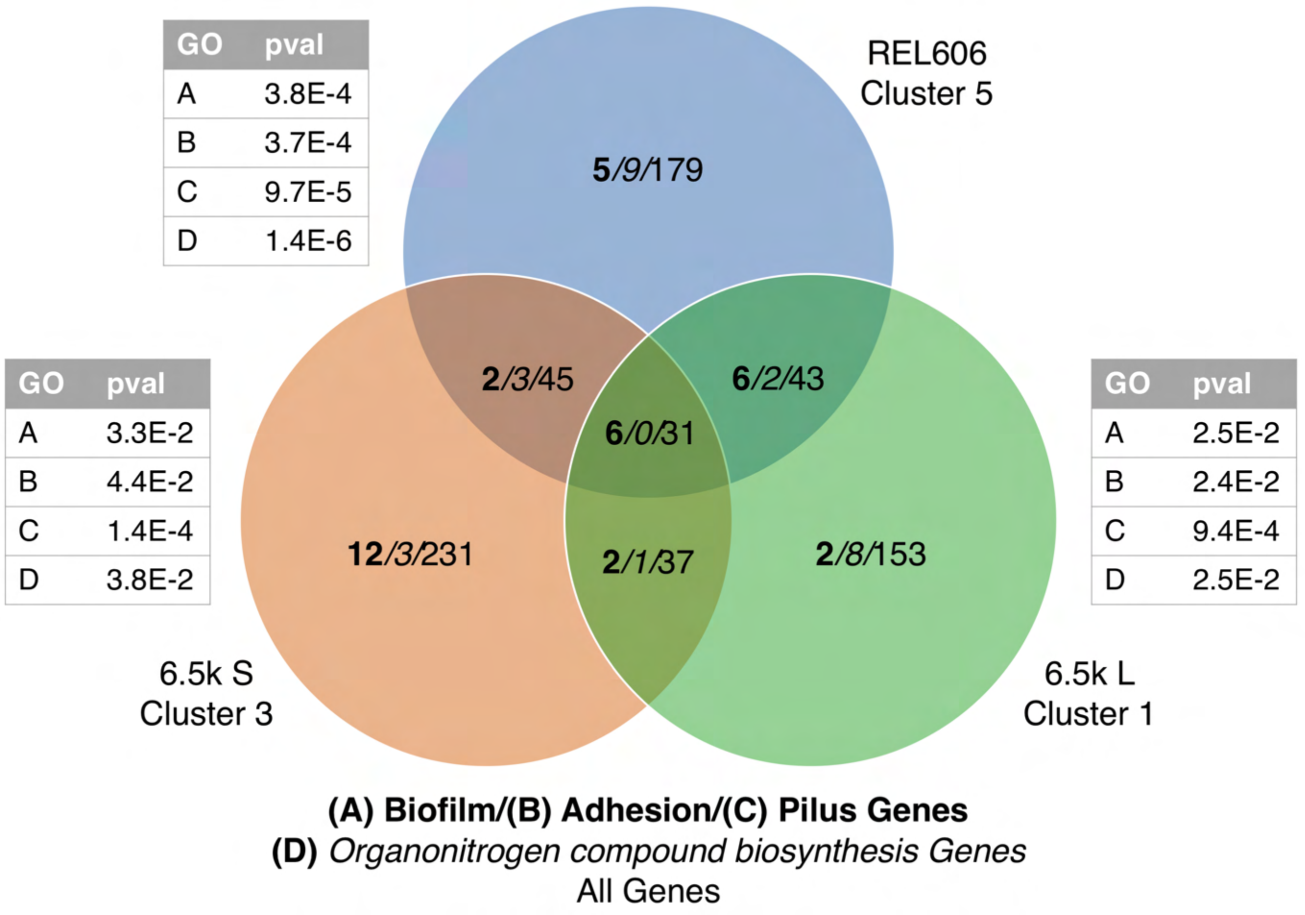

Fig. S18. Biofilm (GO:0043708)/adhesion (GO:0022610)/ pilus organization (GO:0043711)/ organonitrogen compound biosynthesis (GO:1901566) genes tend to appear in the same clusters across genetic backgrounds. P-values are post-FDR correction, using Fisher's exact test.

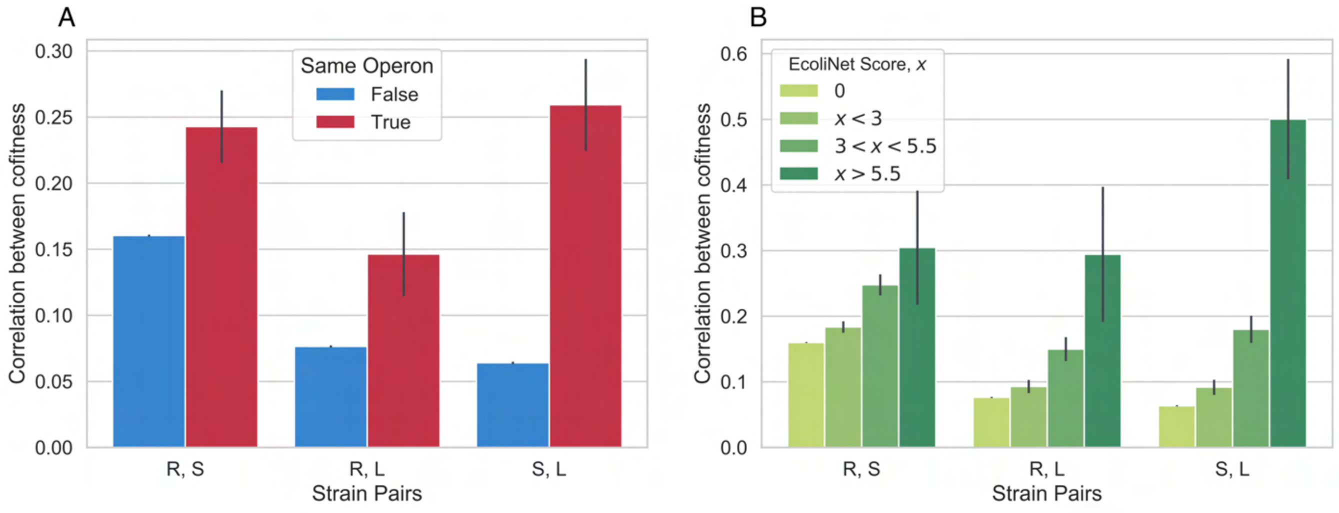

Fig. S19. Correlations between cofitness increase (**A**) when genes are in the same operon and (**B**) with EcoliNet score. A score of 0 indicates that the gene pair is not connected in EcoliNet, i.e. a node distance greater than 1. Error bars represent standard errors. Error bars represent standard errors, computed using a standard bootstrap.

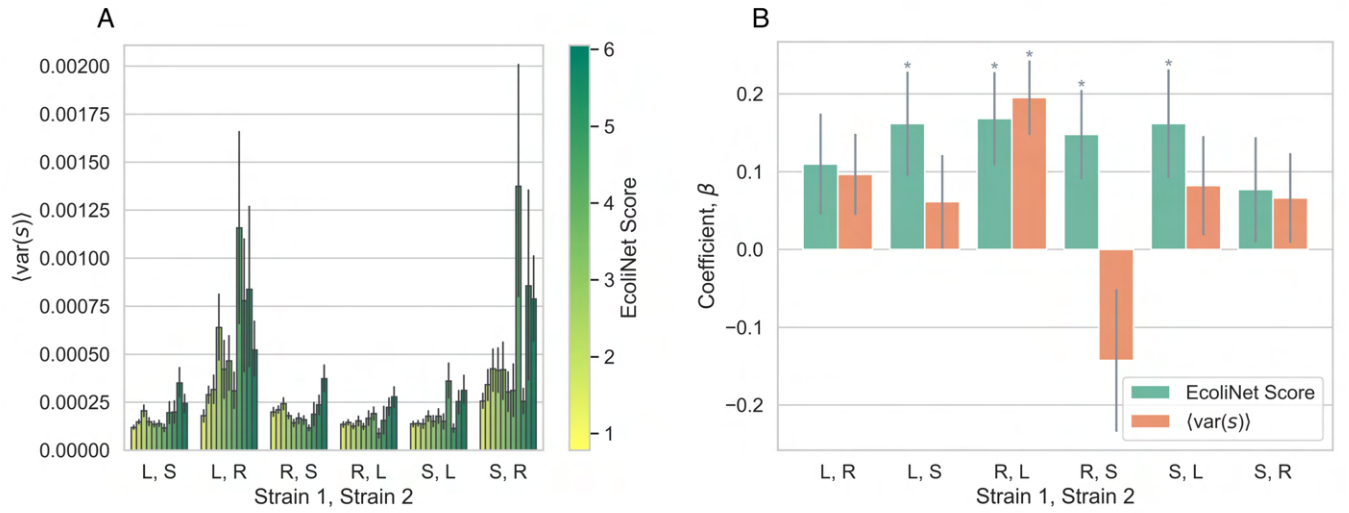

Fig. S20. Variance in fitness effect across environment does not fully explain correlation between EcoliNet score and probability that two genes will be in the same cluster across strains. (**A**) Covariation of EcoliNet scores and variations in fitness effects in some strain pairs. The observed covariation is interesting in and of itself, as it suggests that more strongly interacting genes tend to have a larger variation in fitness effects across environments. Error bars represent standard errors, computed using a standard bootstrap. (**B**) A standard multiple logistic regression with both fitness variance (in strain 2) and EcoliNet score as covariates, with response variable as the probability two genes are in the same cluster in strain 2, if they were together in strain 1. For most strain pairs, the regression reveals that the correlation reported in Figure 4D still holds after controlling for variation in fitness effects. P-values were obtained from the linear model and FDR-corrected, * $p < 0.05$. See section 4.2 for model details. Error bars represent standard errors, computed using a standard bootstrap.

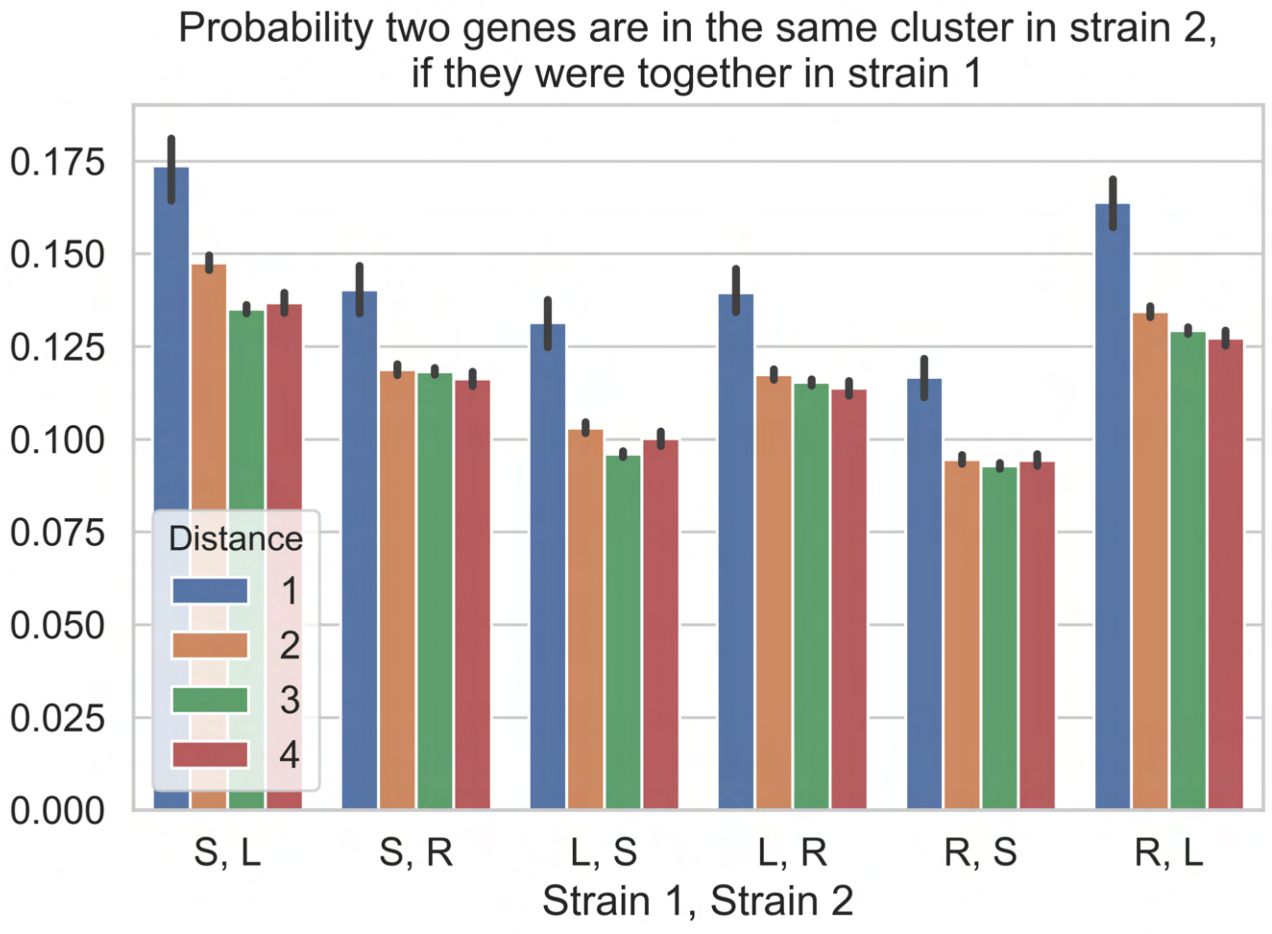

Fig. S21. Shortest distance between genes in EcoliNet predicts if genes stay correlated across genetic background. Error bars represent standard errors, computed using a standard bootstrap.

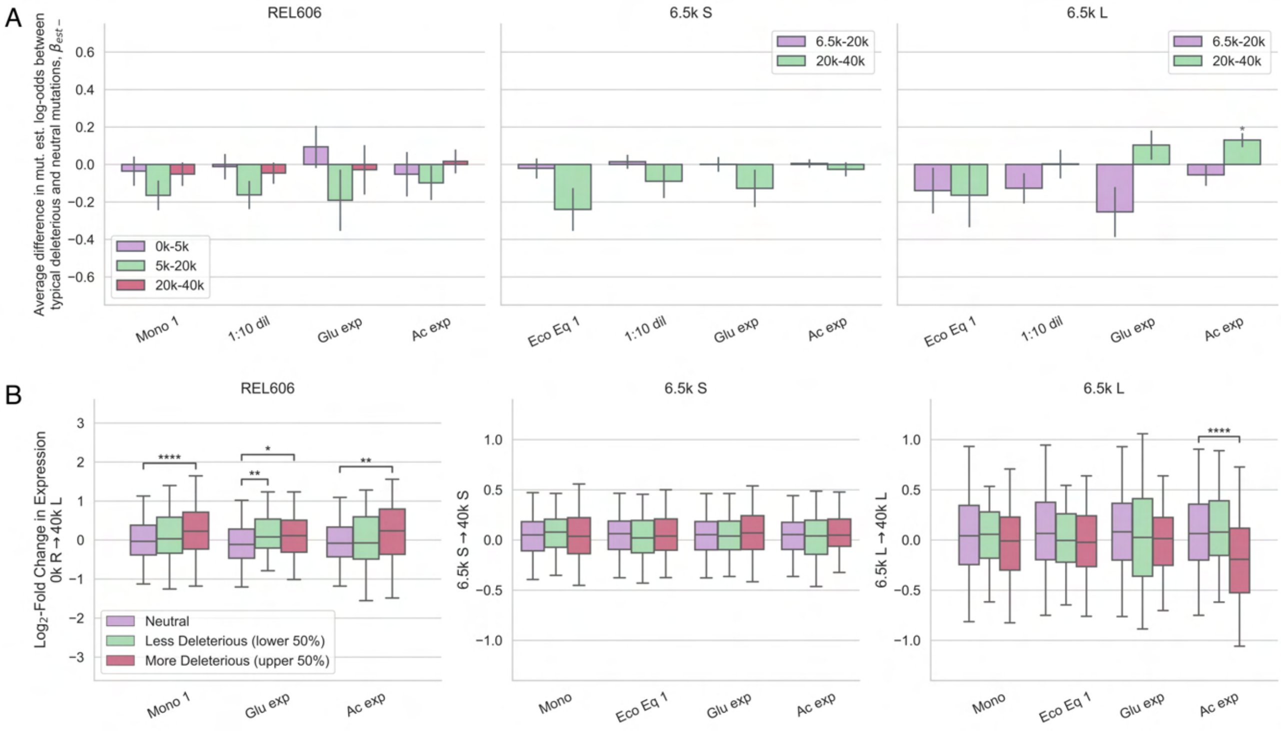

Fig. S22. Relationship between deleterious mutations and evolutionary outcomes. (**A**) Deleterious fitness effects generally do not predict which genes will mutate, with the one exception that L seems more likely than random to get mutations in genes with deleterious acetate knockout fitness. Error bars represent standard errors, which were calculated as detailed in methods section 4.3. FDR-corrected p-values were obtained from the logistic regression model. (**B**) In REL606, deleterious knockout fitness effects are predictive of increased gene expression across all tested environments. The center line of the boxplot is the median; the limits of the box represents the first and third quartiles; the whiskers extend to one times the inter-quartile range; outliers are not shown. FDR-corrected Mann-Whitney U tests were used to compare conditions. Asterisks denote coefficients/comparisons that are significantly different than 0 (* $p < 0.05$, ** $p < 0.01$, *** $p < 0.001$, **** $p < 0.0001$).

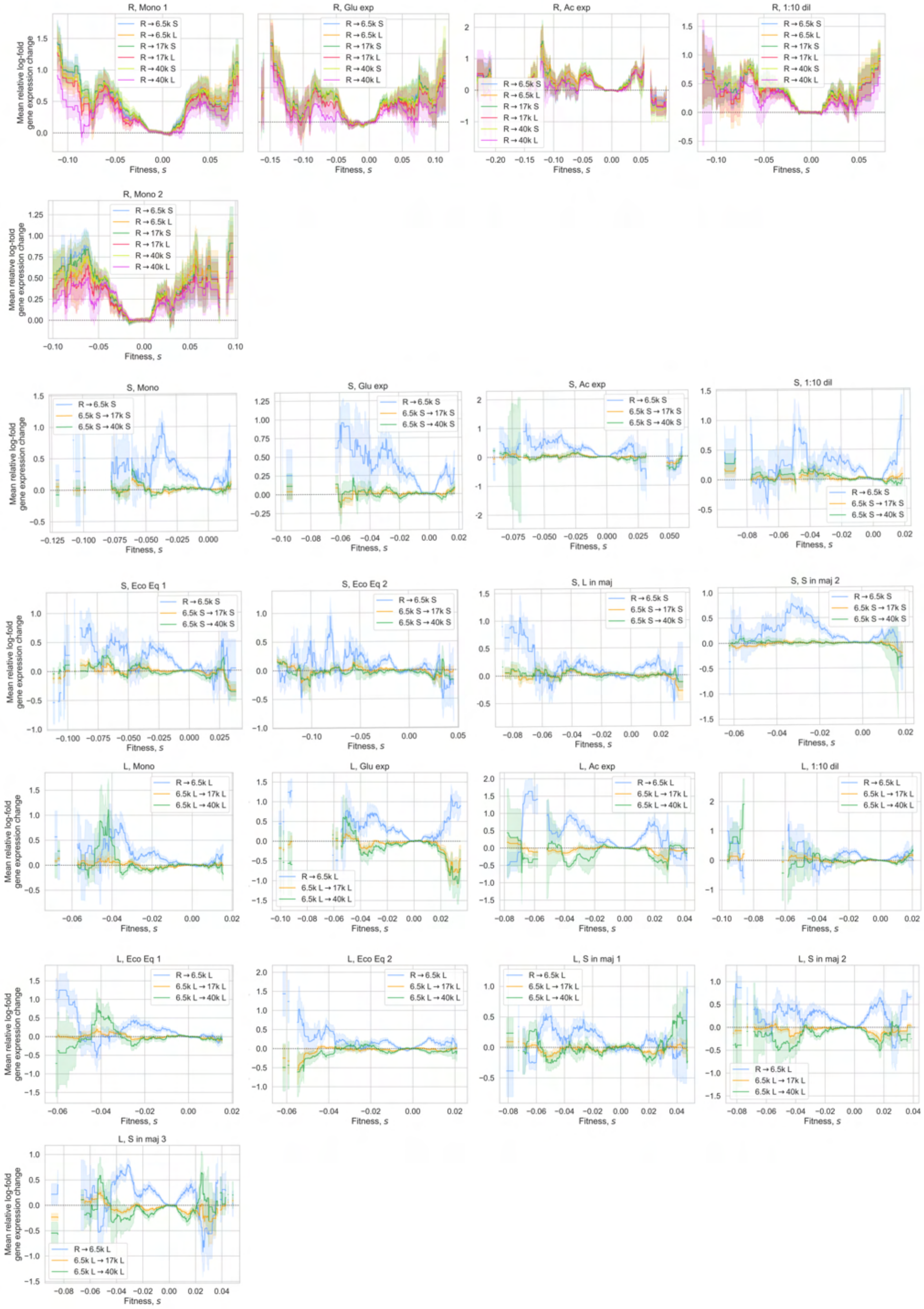

Fig. S23. Relationship between fitness effects and log-fold gene expression change for all experiments, relative to the average change for neutral knockouts. Lines show mean gene expression change as a function of fitness effect ($\pm$ standard error), with a ± 0.01 moving average.

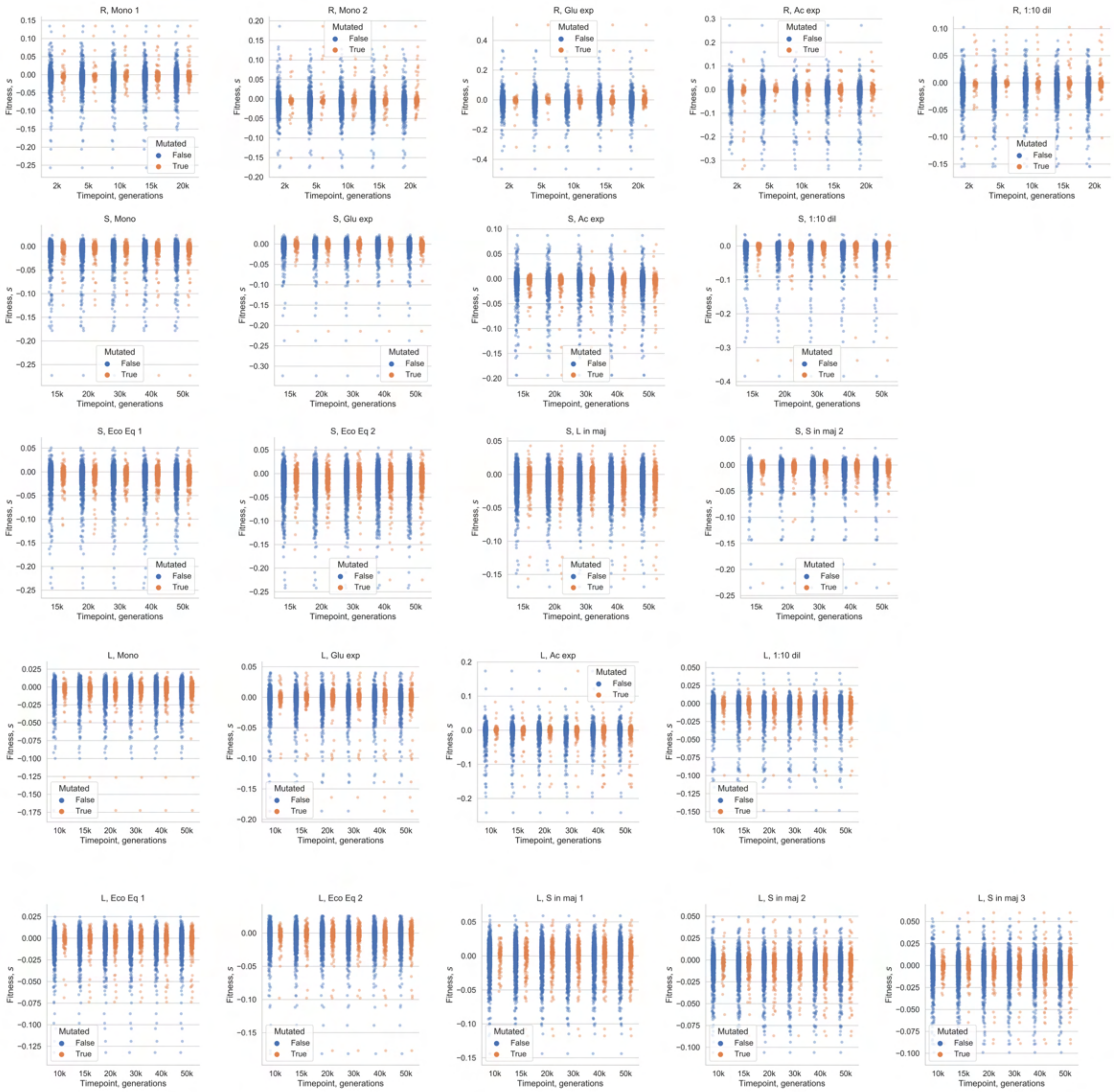

Fig. S24. Establishment of a mutation in a gene by its knockout fitness.

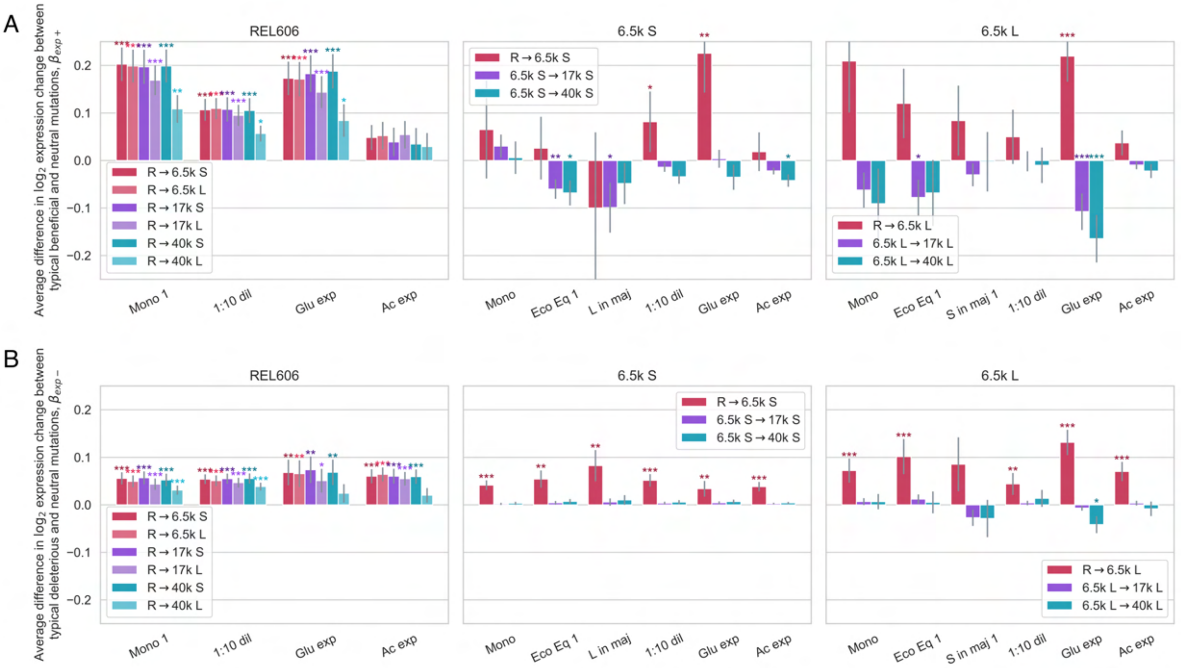

Fig. S25. Linear model to explore correlation of gene expression changes with knockout fitness effects, comparing neutral to (a) beneficial and (b) deleterious knockouts. FDR-corrected p-values were obtained from the regression models detailed in methods section, 4.4. Asterisks denote coefficients that are significantly different than 0 (FDR correction; * $p < 0.05$, ** $p < 0.01$, *** $p < 0.001$). Error bars represent standard errors from the linear model.

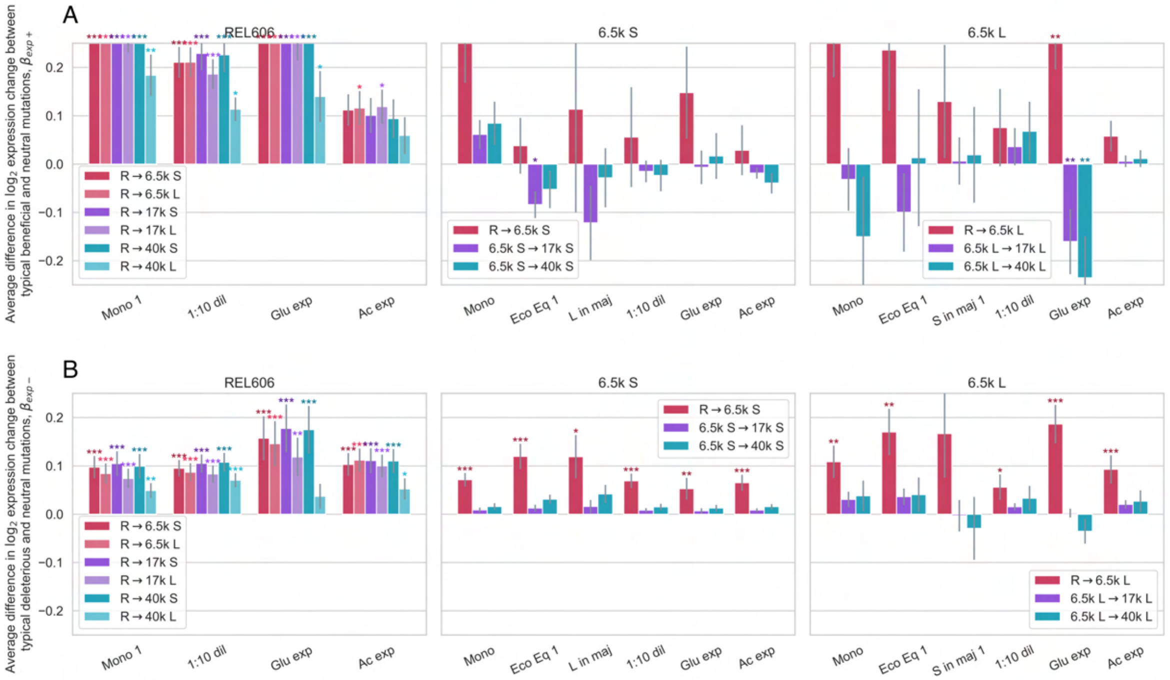

Fig. S26. Restricting analysis of Figure S25 to exclude poorly expressed genes (bottom 50%) does not qualitatively change results of analysis, when comparing neutral to (a) beneficial and (b) deleterious knockouts. FDR-corrected p-values were obtained from the regression models detailed in methods section, 4.4. Asterisks denote coefficients that are significantly different than 0 (FDR correction; * $p < 0.05$, ** $p < 0.01$, *** $p < 0.001$). Error bars represent standard errors from the linear model.

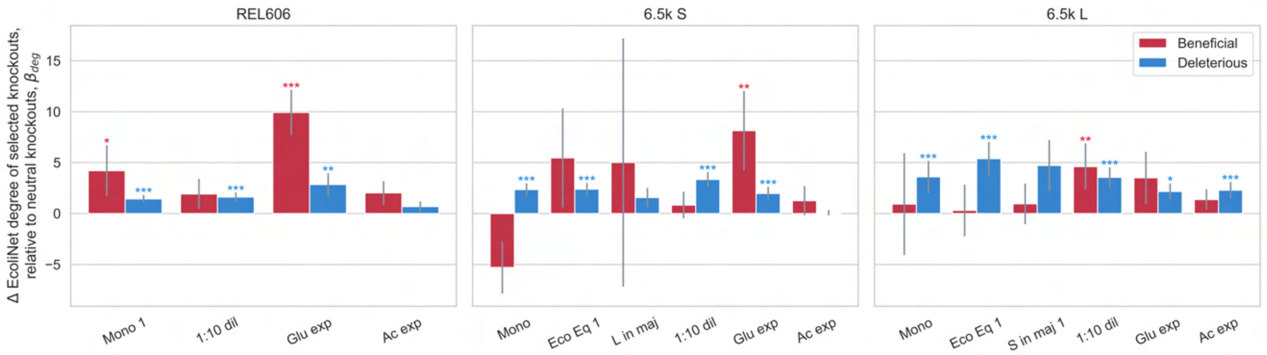

Fig. S27. Fitness effects predict EcoliNet node degree. Deleterious knockouts across environments are more likely to have a high degree compared to neutral knockouts. The same general pattern appears for beneficial knockouts, although less clearly. Linear model fit with ordinary least squares; normalized analogous to model in section 4.4. FDR-corrected p-values were obtained from the linear model. Asterisks denote coefficients that are significantly different than 0 (* $p < 0.05$, ** $p < 0.01$, *** $p < 0.001$). Error bars represent standard errors from the linear model.

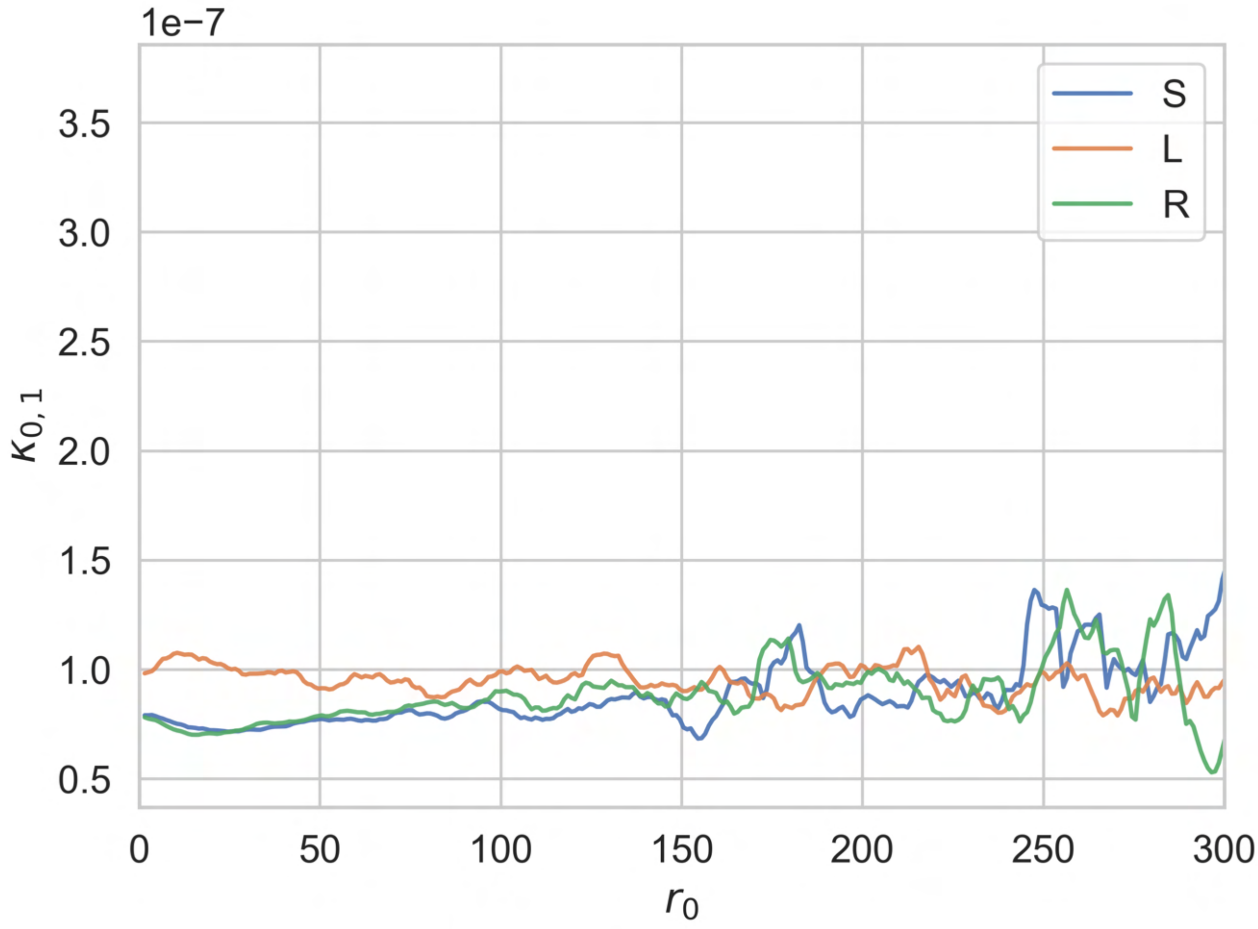

Fig. S28. The measured noise parameter $\kappa_{j,k}$ is consistently approximately constant as a function of initial number of barcode reads. Data is from S/L/REL606 monoculture experiments, replicate 1. Curves are smoothed with a moving average, ± 2 reads.

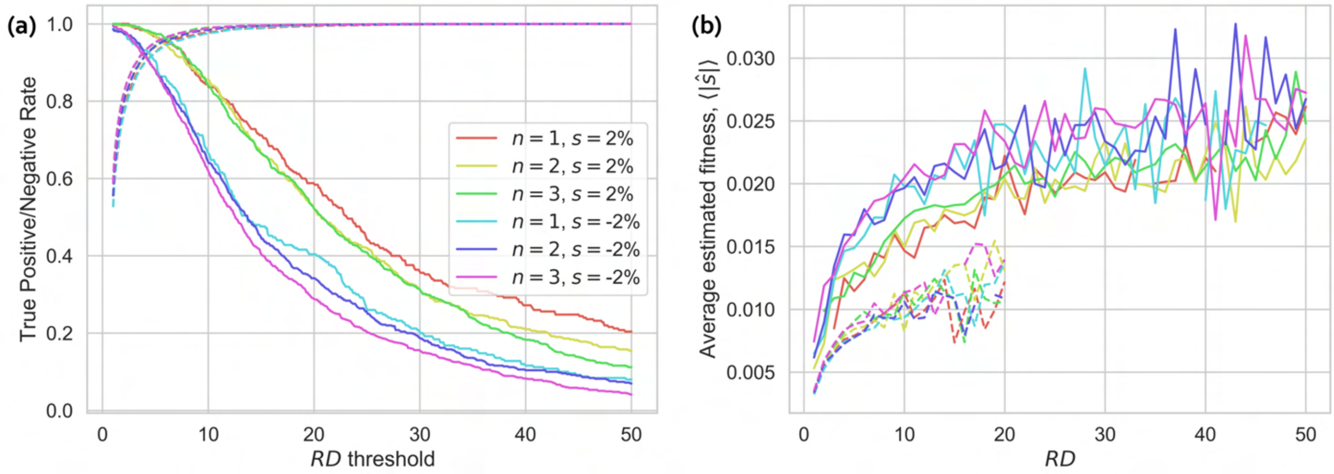

Fig. S29. (a) Detection of selected, outlier barcodes in otherwise neutral genes. Dotted lines are the true negative rate, solid lines are the true positive rate. (b) Average inferred fitness (ie apparent fitness, differing from the true fitness by fluctuations) of barcodes with different RDs. Dotted lines are from neutral barcodes, solid lines are outlier barcodes. 'Neutral' barcodes with $RD \approx 6$ have sufficiently large fluctuations to have trajectories that appear to have a 1% deviation from neutrality.

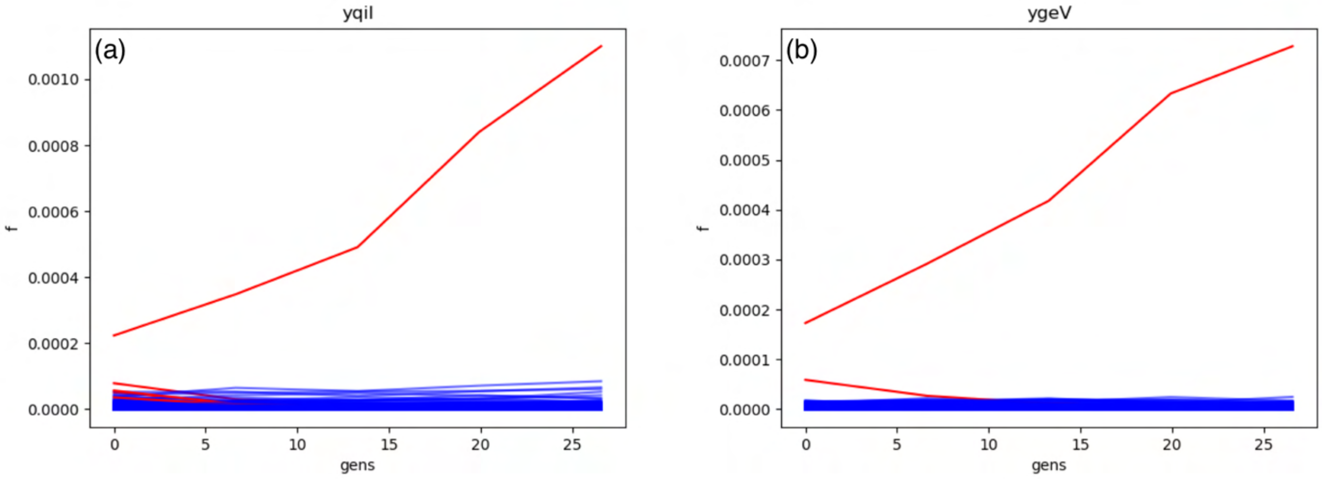

Fig. S30. Examples of high-abundance outlier barcodes detected in otherwise neutral genotypes, (a) *yqiI* and (b) *ygeV* knockouts. Red barcodes were called as outliers. Examples taken from an experiment with the 6.5k S library in co-culture with L at the equilibrium frequency (Eco Eq 2).

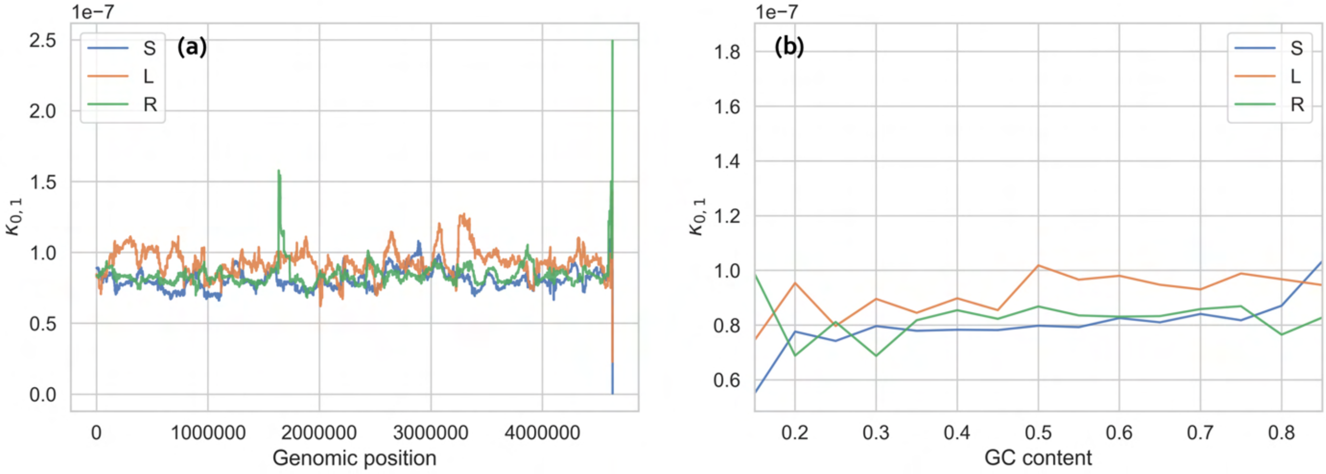

Fig. S31. The measured noise parameter $\kappa_{j,k}$ does not vary systematically over (a) genomic position, or (b) barcode GC content, indicating that these factors do not measurably bias barcode frequency measurements. Data is from S/L/REL606 monoculture (1) experiments, replicate 1.
