# Supplementary material for: Quantifying the local adaptive landscape of a nascent bacterial community
Source: Nat Commun. 2023 Jan 16;14:248. doi: 10.1038/s41467-022-35677-5 (PMC9842643; doi:10.1038/s41467-022-35677-5)
Supplement: Supplementary file 2 — Reporting Summary [file 41467_2022_35677_MOESM2_ESM.pdf]

nature portfolio

Corresponding author(s): Oskar Hallatschek

Last updated by author(s): Nov 28, 2022

Reporting Summary

Nature Portfolio wishes to improve the reproducibility of the work that we publish. This form provides structure for consistency and transparency in reporting. For further information on Nature Portfolio policies, see our Editorial Policies and the Editorial Policy Checklist.

Statistics

For all statistical analyses, confirm that the following items are present in the figure legend, table legend, main text, or Methods section.

n/a	Confirmed	
☐	☒	The exact sample size (*n*) for each experimental group/condition, given as a discrete number and unit of measurement
☐	☒	A statement on whether measurements were taken from distinct samples or whether the same sample was measured repeatedly
☐	☒	The statistical test(s) used AND whether they are one- or two-sided<br>*Only common tests should be described solely by name; describe more complex techniques in the Methods section.*
☐	☒	A description of all covariates tested
☐	☒	A description of any assumptions or corrections, such as tests of normality and adjustment for multiple comparisons
☐	☒	A full description of the statistical parameters including central tendency (e.g. means) or other basic estimates (e.g. regression coefficient) AND variation (e.g. standard deviation) or associated estimates of uncertainty (e.g. confidence intervals)
☐	☒	For null hypothesis testing, the test statistic (e.g. *F*, *t*, *r*) with confidence intervals, effect sizes, degrees of freedom and *P* value noted<br>*Give P values as exact values whenever suitable.*
☒	☐	For Bayesian analysis, information on the choice of priors and Markov chain Monte Carlo settings
☒	☐	For hierarchical and complex designs, identification of the appropriate level for tests and full reporting of outcomes
☐	☒	Estimates of effect sizes (e.g. Cohen's *d*, Pearson's *r*), indicating how they were calculated

Our web collection on statistics for biologists contains articles on many of the points above.

Software and code

Policy information about availability of computer code

Data collection	No software/code was used to collect data
Data analysis	All code used to process and analyze the data is available in the following github repository: https://github.com/joaoascensao/S-L-REL606-BarSeq. Analyses were primarily conducted in Python 3.6.8, along with the following python packages: seaborn 0.11.2, matplotlib 3.0.2, numpy 1.19.5, scipy 1.5.4, scikit-learn 0.20.1, pandas 0.21.0, python-levenshtein 0.12.0, goatools 0.9.5, statsmodels 0.11.0, networkx 2.4. For just the gene expression analysis, we used R version 3.5.1 and limma 3.16.

For manuscripts utilizing custom algorithms or software that are central to the research but not yet described in published literature, software must be made available to editors and reviewers. We strongly encourage code deposition in a community repository (e.g. GitHub). See the Nature Portfolio guidelines for submitting code & software for further information.

Data

Policy information about availability of data

All manuscripts must include a data availability statement. This statement should provide the following information, where applicable:

- Accession codes, unique identifiers, or web links for publicly available datasets
- A description of any restrictions on data availability
- For clinical datasets or third party data, please ensure that the statement adheres to our policy

Glycerol stock copies of the REL606, 6.5k S, and 6.5k L Tn5 barcoded libraries are available upon request. Raw sequencing reads have been deposited in the NCBI BioProject database under accession number PRJNA900607. All processed data are available on GitHub, https://github.com/joaoascensao/S-L-REL606-BarSeq

Other datasets referenced may be accessed online: the LTEE time-resolved clonal sequencing data (https://barricklab.org/shiny/LTEE-Ecoli/); the LTEE time-resolved

metagenomic sequencing data (https://github.com/benjaminhgood/LTEE-metagenomic); S and L clonal sequencing data (Plucain et al. (2014) SI); EcoliNet (https://www.inetbio.org/ecolinet/); transcriptomic measurements of LTEE strains (BioProject PRJNA144635).

Field-specific reporting

Please select the one below that is the best fit for your research. If you are not sure, read the appropriate sections before making your selection.

☒ Life sciences ☐ Behavioural & social sciences ☐ Ecological, evolutionary & environmental sciences

For a reference copy of the document with all sections, see nature.com/documents/nr-reporting-summary-flat.pdf

Life sciences study design

All studies must disclose on these points even when the disclosure is negative.

Sample size	Our sample size was primarily determined by the number of barcoded knockouts that ended up in our libraries--we developed a statistical pipeline to account for the effects of unequal numbers of barcodes per knockout.
Data exclusions	No data was excluded.
Replication	All BarSeq experiments were performed with two biological replicates, and each gene had around a median of ~20 independent barcoded knockouts.
Randomization	Randomization was not relevant for our experimental design.
Blinding	Blinding was not relevant or possible for our experimental design, as the same researcher performed and processed the experiments. However, all of the raw data was run through the exact same data processing pipeline.

Reporting for specific materials, systems and methods

We require information from authors about some types of materials, experimental systems and methods used in many studies. Here, indicate whether each material, system or method listed is relevant to your study. If you are not sure if a list item applies to your research, read the appropriate section before selecting a response.

Materials & experimental systems

n/a	Involved in the study	
☒	☐	Antibodies
☒	☐	Eukaryotic cell lines
☒	☐	Palaeontology and archaeology
☐	☒	Animals and other organisms
☒	☐	Human research participants
☒	☐	Clinical data
☒	☐	Dual use research of concern

Methods

n/a	Involved in the study	
☒	☐	ChIP-seq
☒	☐	Flow cytometry
☒	☐	MRI-based neuroimaging

Animals and other organisms

Policy information about studies involving animals; ARRIVE guidelines recommended for reporting animal research

Laboratory animals	Our study did not involve laboratory animals. The only organisms used in this study were Escherichia coli B strains.
Wild animals	Our study did not involve wild animals.
Field-collected samples	Our study did not involve field-collected samples.
Ethics oversight	Our study did not require ethical oversight.

Note that full information on the approval of the study protocol must also be provided in the manuscript.
